# Dry Eye Post-Laser-Assisted In Situ Keratomileusis: Major Review and Latest Updates

**DOI:** 10.1155/2018/4903831

**Published:** 2018-01-28

**Authors:** Eyal Cohen, Oriel Spierer

**Affiliations:** Division of Ophthalmology, Tel Aviv Sourasky Medical Center, Sackler Faculty of Medicine, Tel Aviv University, Tel Aviv, Israel

## Abstract

Dry eye is one of the most common complications occurring after laser-assisted in situ keratomileusis (LASIK), with virtually all patients experiencing some degree of postoperative dry eye symptoms. Enhanced understanding of the pathophysiology and mechanism of dry eye development in addition to preoperative screening of patients who are prone to dry eye is essential for better patient satisfaction and for improving short-term visual outcome postoperatively. This article reviews the latest studies published on LASIK-associated dry eye, including epidemiology, pathophysiology, risk factors, preoperative assessment, and management.

## 1. Introduction

Dry eye following laser in situ keratomileusis (LASIK) is the most common problem faced by refractive surgeons, with virtually all patients experiencing some dry eye after surgery, usually transiently [1]. This side effect has a major contribution to patient dissatisfaction after LASIK [2] and causes frustration for both patients and surgeons. Dry eye was responsible for 19% of referrals to the cornea service in a tertiary hospital due to nonsatisfaction with the results after LASIK [3]. Lower satisfaction rates were reported by patients who had one or more symptoms of dryness or recurrent corneal erosions compared to patients reporting no symptoms, and fewer of these patients were willing to have repeat surgery than patients without dry eye symptoms [4]. From the ophthalmologist's perspective, dry eye demands extensive investment in time to treat, with no easy cure and occasional discrepancy between signs and symptoms [2]. Patients often report ocular discomfort, foreign body sensation, eye fatigue, and irritation, despite having a normal ocular surface. It is generally believed that LASIK induces a greater decrease in tear secretion and corneal sensitivity, with more profound dry eye symptoms than photorefractive keratectomy (PRK) [5–7], although this belief was not supported in some studies [4, 8].

Many patients who seek LASIK surgery can not tolerate contact lenses because of a preexisting dry eye syndrome or dry eye syndrome secondary to long-time contact lens use. It is estimated that 30% of the candidates for refractive surgery have dry eye symptoms and that 50% of the patients have clinical signs of dry eyes prior to surgery [9]. Importantly, preexisting dry eye is a risk factor for severe postoperative dry eye, with lower tear function, more corneal staining, and more severe symptoms [10, 11].

Signs of post-LASIK dry eye include positive staining of the ocular surface, decrease in tear breakup time (TBUT), lower basal tear secretion, positive Schirmer test, and reduced corneal sensitivity [9, 12, 13]. Functional visual acuity can also be influenced [12]. The risk for refractive regression after LASIK was reportedly increased in patients with chronic dry eye, and patients with chronic dry eyes had significantly worse myopic outcomes than those without dry eyes [14].

The exact mechanism by which LASIK causes ocular dryness is not clear, but it is known to be multifactorial. Possible mechanisms include decreased trophic influence on the corneal epithelium due to corneal nerve severing, reduced corneal sensation and blink reflex, damage to the limbal goblet cells caused by the pressure of the suction ring, altered tear film stability due to change in corneal curvature, inflammation, and a toxic effect of topical drops [15]. There is no consensus among researchers regarding the risk factors for prolonged ocular dryness after LASIK; however, patients with preexisting dry eyes apparently are at increased risk [9, 16]. Other possible risk factors include female sex, Asian ancestry, and high myopia [17–19]. Surgical parameters, such as suction time [20], ablation depth [21, 22], and hinge location can also play a role [15, 23]. Artificial tears are the primary treatment of the post-LASIK dry eye [13]. In more severe cases or when dryness becomes chronic, other treatment modalities include cyclosporine A 0.05% (Restasis) [24], autologous serum eye drops [25], and punctal plugs [13, 26].

## 2. Incidence

Estimates of the incidence of dry eye syndrome after LASIK vary widely, but almost all patients will have transient dry eye during the postoperative period [27], with the greatest incidence of dry eye after surgery at one week [22]. One study found that 94.8%, 85.4%, and 59.4% of patients experienced dry eye symptoms at 1 day, 1 week, and 1 month after surgery, respectively [9]. Another report found that dry eye affected approximately 50% of patients at 1 week, 40% at 1 month, 20% at 3 months, and 10–40% at 6 months [22]. In contrast, a very large military study demonstrated that dry eye after LASIK is infrequent and that dry eye requiring punctal plugs was reported in only 0.04% of cases [28].

## 3. Pathophysiology

Most corneal nerve fibers are sensory and derived from the ophthalmic branch of the trigeminal nerve. They enter the cornea at the periphery in a radial fashion parallel to the corneal surface and are located mainly in the anterior third of the stroma. Stromal fibers turn at a 90-degree angle and proceed towards the corneal surface, penetrating Bowman's membrane. They are then divided into several smaller nerves, which again turn at a 90-degree angle and continue parallel to the corneal surface, between Bowman's membrane and the basal epithelial cell layer. They then course obliquely into the more superficial epithelial cell layers where they eventually terminate [29]. Maintenance of a normal ocular surface requires an intact neural loop for the ocular surface-lacrimal gland functional unit [30] since the drive for lacrimal tear production and the blink mechanism are stimulated by the sensory nerves that innervate the ocular surface (cornea and conjunctiva) [31].

As noted earlier, the mechanism by which LASIK causes dry eye is multifactorial [15]. Flap creation and corneal ablation sever most of the nerves that course from the limbus to innervate the stroma and epithelium in the central cornea, which results in a decrease in tear secretion after the surgery. An inhibited feedback loop from the cornea to the lacrimal gland was shown to cause this tear production disturbance [32]. A recent study reported an inverse relationship between reinnervation post-LASIK and dry eye symptoms, suggesting that post-LASIK dry eye is a neuropathic disease [33]. The loss of neural stimuli also increases tear osmolarity by decreasing lacrimal gland protein, electrolyte, and water secretion. Osmolarity may also rise due to an increase in tear film evaporation resulting from the absence of previously worn glasses [5]. Increased tear osmolarity induces ocular surface inflammation by activating stress kinases which alter the ocular surface. In addition, the relative hypoesthesia also leads to a drop in the blink rate, with an increase in the evaporation of the tears.

Reports of punctate epithelial erosions and rose bengal staining with a normal Schirmer test (which indicates normal tear production) have led to the identification of a condition called “laser in situ keratomileusis-induced neurotrophic epitheliopathy” (LINE). In this condition, corneal staining is due to diminished neurotrophic factors released from the severed nerves [34] or from other factors, such as a decrease in the frequency of blinking and an increase in the ocular surface exposure [35]. The condition typically resolves approximately 6 months after the surgery when the corneal nerves tend to reinnervate the flap [34].

It would appear that two different mechanisms are capable of causing dry eye after LASIK. One is a reduction in Schirmer test results that indicates an aqueous tear deficiency. Another is LINE secondary to a reduction of blinking and a decreased release of neurotrophic factors [36]. Treatment for LINE has not yet been adequately defined, and since tear production in cases of LINE is normal, the need for punctal occlusion is open to questions [37]. A third possible mechanism is called the “phantom cornea.” LASIK causes damage to corneal nerves, giving rise to aberrant spontaneous and stimulus-evoked nerve impulse firing. It was speculated that these abnormal sensory discharges are read by the brain as ocular surface dryness. A “phantom cornea” could explain the fact that many patients experience dryness after the surgery despite the often modest disturbances of tear secretion and ocular surface staining. Other factors contributing to post-LASIK dryness include inflammatory response to surgery with release of cytokines and immune mediators [9, 38–40] and reduction in goblet cell density [41–44]. After LASIK, the lids move over a new ocular surface which can cause surface tension alteration and tear film layer instability [23]. Frequent post-LASIK instillation of eye drops which contain preservatives may induce a toxic effect on the conjunctiva and cornea with accompanying dryness [5, 45].

## 4. Risk Factors for Significant or Prolonged Dry Eye

The most significant risk factor for developing severe ocular dryness after LASIK surgery is preexisting dry eyes [11, 13, 45]. Preoperative tear volume may affect the recovery of the ocular surface after LASIK, increasing the risk of chronic dry eye [16]. A Schirmer test value of less than 10 mm before surgery is a specific indicator for experiencing dry eye symptoms after LASIK [9]. Ocular surface staining, low corneal sensation, and dry eye symptoms before LASIK are correlated with higher risk for chronic dry eyes after LASIK [14]. Subjects requiring higher refractive correction have an increased risk for developing dry eyes [17, 19, 46]. Patients undergoing LASIK for high myopia (range: −9.10 to −14.00 D) reported ongoing dry eye symptoms 2–5 years after surgery [19].

It is possible that the parameters in the LASIK treatment itself are important in the risk for developing dry eye. For example, suction time and the diameter of the ablation zone affect corneal sensitivity and the incidence and severity of dry eye [20, 47]. A prospective randomized clinical trial found that dry eye was associated with the level of preoperative myopia, ablation depth, and flap thickness [22]. While some investigators have shown that ablation depth is an important factor in the temporary decrease of corneal sensitivity and its recovery [21, 46], others stated that ablation depth [15, 48] or flap thickness [20, 48] do not affect ocular dryness after surgery [2]. Recurrence of dry eye signs and symptoms may be expected with LASIK enhancement [34]; however, patients who had flap lifting and underwent retreatment did not complain of dry eye symptoms and their tear functions were not compromised after the procedure [49].

A long-term history of wearing contact lenses before LASIK may contribute to delayed recovery in tear secretion and corneal sensitivity after surgery [39, 50]. Other factors that may contribute to post-LASIK dry eyes include previous blepharoplasty, lagophthalmos [45], and diabetes mellitus [51]. A genetic influence was also observed with polymorphism in the thrombospondin 1 (*THBS1*) gene that was found to be associated with postrefractive surgery chronic ocular surface inflammation [52].

Asian ancestry has a higher prevalence of chronic post-LASIK tear dysfunction and dry eye symptoms; according to one study, 28% of Asian patients suffered from dry eye symptoms compared to 5% among Caucasian patients post-LASIK surgery [18]. Contributing factors may include eyelid and orbital anatomy, tear film parameters, and blinking dynamics, as well as higher rates of attempted refractive corrections in Asian eyes [18]. Some reports indicated that female sex is a risk factor for post-LASIK dry eyes [17, 53], while others found that premenopausal females had less dry eye than males post-LASIK [2]. More recent data now suggest that neither gender nor age influences the risk for dry eyes post-LASIK [2, 48].

### 4.1. Hinge Location

The few studies that tried to determine whether corneal sensation and the post-LASIK dry eye state are affected by hinge characteristics [15] produced conflicting results. The hinge provides a conduit for corneal innervation. The corneal nerves that enter through the hinge are preserved, maintaining corneal sensation in this area [54]. One prospective, randomized study of 52 consecutive patients (104 eyes) found that the loss of corneal sensation and the presence of dry eye syndrome were greater in eyes with a superior-hinge flap than in eyes with a nasal-hinge flap: corneal sensation returned to normal at 6 months post-LASIK in the eyes with nasal-hinge flaps, but it did not return to preoperative levels even by 6 months in corneas with a superior-hinge flap [23]. Similarly, another study found that the TBUT and Schirmer test results were higher in the nasal hinge group than in the superior hinge group at 6 months post-LASIK [55]. Recovery of corneal sensitivity was most rapid in a flap with a nasally placed hinge [56]. However, a number of studies showed that hinge position, that is, superior versus nasal, had no significant effect on corneal sensation or dry eye parameters [15, 32, 57, 58]. A prospective study that compared between a nasal hinge flap in one eye and a superior hinge flap in the fellow eye did not find any difference in corneal sensation, basic secretion test, tear film breakup time, conjunctival and corneal staining, or responses to a subjective questionnaire at 3 and 6 months post-LASIK [32]. Moreover, one study even found that the decrease in corneal sensitivity in LASIK patients was significantly greater in those with a nasal hinge than in those with a superior hinge [59]. This controversy is partially associated with the lack of agreement about corneal innervation. Traditionally, it was thought that the long ciliary nerves enter the cornea at the 3 o'clock and 9 o'clock sites, with greater susceptibility to corneal nerve damage with vertically hinged than with horizontally hinged flaps [23]. However, the validity of this model has been brought into question since in vivo confocal microscopy studies showed that the subbasal nerve fibers are in a 6- to 12-hour (superior-to-inferior) orientation [29].

A meta-analysis that aimed to resolve this issue showed that a horizontal-hinge flap causes less loss of sensation than the vertical-hinge flap and that the difference was significant at 3 months postoperatively [27]. The TBUT value was significantly larger in the horizontal-hinge flap than in the vertical-hinge flap at 1 and 3 months postoperatively. In addition, a significantly greater percentage of patients with corneal fluorescein staining had a superior-hinge flap compared to those with a nasal-hinge or a temporal-hinge flap at 1 and 3 months after surgery but not after 6 months; Schirmer's test values were also higher in the horizontal-hinge group, but the difference did not reach a level of statistical significance at any of the postoperative periods. Those authors concluded not only that hinge location may have some effect on corneal sensation and dry eye syndrome after LASIK at the early postoperative period but also that there was no significant difference between the groups at 6 months after surgery [27]. Whether other features of flap structure, such as the width of the flap [54, 60] or the hinge angle [15] have any effect on dryness awaits further investigation.

### 4.2. Femtosecond Laser versus Microkeratome

The incidence of LASIK-associated dry eye is higher in patients who had LASIK with microkeratome than in patients in whom the surgery was done with the femtosecond laser [20]. The high pressure induced by the suction ring during LASIK may damage the conjunctival goblet cells, thereby compromising the mucin layer of the tear film [1]. Decreased conjunctival sensitivity, which was observed even by 18 months post-LASIK, may be due to trauma to the perilimbal conjunctival nerves by the microkeratome suction ring [39]. Impression cytology showed a greater reduction in goblet cell populations after microkeratome than those after femtosecond laser, probably because of differences in the length of time that the suction ring exerted pressure on the conjunctiva. Thinner flaps associated with the femtosecond laser may cause less postsurgery dryness due to less damage to the afferent sensory nerves in the anterior stroma [20, 60]. In a recent prospective study [61], the eyes were randomized for flap creation by femtosecond laser or microkeratome. The mean central flap thickness was lower in the femtosecond group (111 *μ*m) compared to the microkeratome group (131 *μ*m). It should be noted that other investigators could not find any differences between femtosecond laser and mitcrokeratome in terms of corneal sensitivity, Schirmer testing, TBUT, conjunctival and corneal stainings, and subjective symptoms at 1 week and 1, 3, and 6 months postoperatively [61].

## 5. Evaluation of Patients and Ocular Surface Prior to LASIK

Prior to surgery, LASIK candidates should be carefully questioned about dry eye symptoms and undergo a detailed evaluation of the external ocular surface (Table 1). The candidate should be directly questioned regarding feelings of dryness, irritation, tired eyes, and vision fluctuations in conjunction with blinking [62]. The Ocular Surface Disease Index (OSDI) questionnaire helps collecting a detailed preoperative information regarding dry eye symptoms and signs. The clinician should look for the signs of dry eye syndrome, including conjunctival injection, punctate keratitis, reduced tear meniscus, tear film debris, abnormal TBUT, abnormal Schirmer testing, and distorted mires on corneal topography [30]. The Schirmer test is easy to perform in a clinical setting, and it remains the mainstay in the clinical diagnosis of dry eye despite its inaccuracy (with only 25% sensitivity and 90% specificity) [63] calculating the ocular protection index (OPI) by measuring the TBUT and interblink interval (IBI) and dividing TBUT by the IBI; a score higher than one predicts a better protected ocular surface [64]. Additional examinations include tear osmolarity, tear mucin measurement, goblet cell count, tear lysozyme measurement, and lactoferrin measurement, but these tests are rarely done in clinical practice.

Caution is needed when evaluating patients with dry eyes. A trial of dry eye treatment is needed before performing LASIK surgery, and the surgery can be scheduled if patients respond well to treatment [30]. Refractive outcomes in patients with preoperative dry eyes tend to be good, and although patients may experience a transient decrease in visual acuity after surgery, there is generally no significant difference in refractive outcomes in patients with dry eyes who are diagnosed before or after LASIK [62]. Patients with severe dry eye symptoms and persistent corneal staining or decreased vision due to ocular surface disease, however, may not be suitable for LASIK [30].

## 6. Symptoms and Signs

When diagnosing a post-LASIK dry eye state, the most important factor is the anamnesis since no single test is sufficient to diagnose dry eye [15] and signs often do not correlate with the symptoms. Neither the Schirmer tear test nor the rose bengal stain score correlates well with symptoms of ocular dryness [65]. Subjective questionnaires, such as the Ocular Surface Disease Index questionnaire, can also be used [58].

Patients may complain of irritation, foreign body sensation, pain, grittiness, and photophobia. Compromised tear function may induce a disruption of ocular surface integrity, which disturbs clear vision [66] and causes fluctuation in vision in approximately 10% of patients [11]. Functional visual acuity is defined as the visual acuity after 10 seconds of keeping the eye open, and it was found to be decreased after LASIK surgery [12]. If patients keep their eyes open for a longer time than the TBUT, functional visual acuity may be compromised due to irregular astigmatism induced by tear film instability. This may interrupt daily activities, such as reading and driving. The postoperative dry eye symptoms are temporary in their nature and more pronounced during the first month, usually lasting fewer than 12 months after the surgery [48].

## 7. Evaluation of Ocular Surface and Tear Production

Corneal fluorescein staining is at its peak at 1 week post-LASIK. Many of these patients do not have feelings of discomfort, probably because the flap has sensory denervation [34]. The condition tends to be self-limiting, resolving approximately 6–12 months after LASIK [34, 39, 58]. The tear flow may be hampered, as demonstrated by decreased Schirmer and basal secretion test values [9, 66]. Tear secretion returns to its preoperative values only after 9 months [50]. TBUT was reported to decrease as early as the first day after the surgery [66], and tear film stability is reestablished with normal TBUT values by one month after surgery [9]. Other findings in post-LASIK eyes may include elevated tear osmolarity [8, 67], increased tear film levels of MMP-9 [40], and a thin tear lipid layer [68]. Fine lines in a lattice pattern, which are folds in the epithelium or Bowman's membrane and associated with dry eyes, were also reported after LASIK [69]. Corneal surface regularity, as evaluated by topography, can also be affected by a dry eye state after LASIK [39]. Evaluation of tear meniscus after LASIK surgery by anterior segment spectral-domain optical coherence tomography (SD-OCT) found that both upper and lower tear menisci decrease after surgery and recover gradually up to 20 months post-LASIK [70, 71].

In contrast, other studies showed that parameters indicating dry eye do not change after LASIK, among them corneal staining, tear breakup time, Schirmer test, tear meniscus height, tear thinning time, osmolarity, and topography [34, 58, 72, 73].

Lastly, the blink rate was shown to be decreased at 3 months, 6 months, and 1 year after LASIK. The drive for normal blinking starts by a sensory branch of the trigeminal nerve (corneal sensitivity) as the afferent arm and a motor branch of the facial nerve as the efferent arm. Blinking may be suppressed when sensory innervation is severed by flap formation, and it may also be modified by other environmental and psychological factors [66].

## 8. Recovery of Corneal Innervation and Sensitivity after LASIK

Corneal sensitivity at the central and paracentral areas [21] is known to decrease significantly after LASIK, and it is lowest at 1 to 2 weeks postoperatively [74]. Recovery is slow: it takes longer than 6 months [46] and appears to be complete after 12 months [59], although it could take 18 months and more [39]. Corneal sensitivity may not only recover to preoperative levels but could also improve after LASIK due to the cessation of contact lens wearing [66].

In confocal microscopy, the corneas of rabbits demonstrated epithelial and basal epithelial/subepithelial nerve disappearances from the flap (excluding its hinge) after LASIK [75]. The density of these nerves was restored to near normal 2.5 months postsurgery. In humans, the subbasal nerve fiber density showed a gradual recovery after LASIK, although the process took longer than 6 months [74] and in many cases longer than 24 months [76] or even 60 months compared to 24 months after PRK [77]. The regenerated nerve fibers were thinner, more curved, and showed abnormal branching in nearly all patients, but normal sensation was regained independently of normal subbasal nerve anatomy [78]. In general, recovery of corneal sensation is not correlated with regeneration of corneal nerves, since corneal sensation recovered to preoperative levels within the first year after surgery while corneal nerve morphology continued to be abnormal [62].

## 9. Management

The therapeutic approach is to stabilize the tear film, increase lubricity, increase aqueous production, and, overall, create a more normal tear environment. Ocular surface management significantly minimized LASIK-induced decreases in goblet cell density and reduced dry eye symptoms [41]. Intensive dry eye treatment may improve the refractive outcome and alleviate the need for enhancement surgery [14]. Artificial tears are the key treatment of the post-LASIK dry eye [13], especially the preservative-free type [79, 80]. Given that all patients have some degree of dry eye disease after LASIK, all patients are treated with preservative-free artificial tears for a time period at the discretion of the surgeon. In cases where dryness is more severe or prolonged, further therapeutic tools should be considered. One prospective, randomized study found that TBUT and rose bengal staining were significantly improved after LASIK in patients who received autologous serum eye drops, whereas no change in these parameters was reported in the patients who received only artificial tears [81]. Topical cyclosporine A 0.05% (Restasis) may also be effective for treating LASIK-induced dryness, inflammation, and neurotrophic epitheliopathy. A retrospective study that compared patients in whom a standard postoperative eye drop regimen was followed with patients in whom cyclosporine A 0.05% (Restasis) was added to the standard regimen for 12 weeks found that cyclosporine A 0.05% was associated with overall better and faster recovery of uncorrected visual acuity [24]. The mean refractive spherical equivalent in eyes treated with cyclosporine A 0.05% was significantly closer to the intended target at 3 and 6 months after LASIK than that in eyes treated solely with artificial tears. Also, a greater percentage of cyclosporine A 0.05%-treated eyes was within +/−0.5 D of the refractive target 3 months after surgery than artificial tear-treated eyes [82]. Likewise, cyclosporine A 0.05% drops were likely to be beneficial in patients with preexisting dry eye who are considering LASIK surgery [11, 13, 20, 34].

A new formulation of cyclosporine 0.1% cationic emulsion (IKERVIS) was introduced recently in Europe for the treatment of severe keratitis in adult patients with dry eye disease, which has not improved despite treatment with tear substitutes [83]. Studies examining the effectiveness of the drug for treatment of dry eye post-LASIK have not yet been published.

Tacrolimus, a macrolide produced by *Streptomyces tsukubaensis*, is another immunosuppressive agent, but its immunosuppressive potential is higher than that of cyclosporine [84]; tacrolimus is mainly used for graft versus host disease with severe dry eye or Sjögren syndrome-related dry eye [85, 86]; studies for dry eye post-LASIK is not yet conducted. A promising novel medication for dry eye treatment is the Diquafosol ophthalmic solution 3% which stimulated fluid secretion from conjunctival epithelial cells and mucin secretion from the conjunctival goblet cells. A combined therapy of Diquafosol tetrasodium and sodium hyaluronate improved visual performance and alleviated dry eye symptoms post-LASIK [87].

Biologic molecules such as lubricin (proteoglycan-4), recombinant human nerve growth factor, tumor necrosis factor *α*-stimulated gene/protein-6, interleukin-1 receptor antagonist, antitumor necrosis factor-*α* therapy, and anti-interleukin-17 hold a promising future in the treatment of dry eye disease. However, although many biologics have been thoroughly investigated in animal models, human studies remain scarce and well-designed human trials are required to further assess their therapeutic role [88].

Punctal plugs are a safe, effective, and reversible method of preserving aqueous and artificial tears on the ocular surface in order to reduce the signs and symptoms of dry eye [13]. Punctal plugs can improve not only the symptoms of dry eye and tear function but also the quality of vision [26, 89]. In post-LASIK patients, punctal occlusion was shown to improve both uncorrected visual acuity and functional visual acuity [26]. In addition, it may reduce lower and higher order aberrations by changing the curvature, surface tension, volume, and dynamics of the tear film [89].

Treatment for dry eye has included topical steroids [80], bandage contact lenses [45], and even temporary tarsorrhaphy [80]. The use of prosthetic replacement of the ocular surface has also been described [90]. Additional treatments that may have a positive impact on post-LASIK ocular dryness include nutritional supplements, such as omega-3 essential fatty acids [30], tricyclic antidepressants [91], and eye warming devices [92].

Platelet-rich plasma showed evidence for promoting the subbasal nerve plexus regeneration after LASIK. However, it provided no positive effect on the recovery of corneal sensitivity, probably due to the limited bioavailability of growth factors in the corneal stroma when platelet-rich plasma is topically administered [93]. Topically applied nerve growth factor may play a future role in accelerating corneal reinnervation after LASIK. Rabbits' eyes that were treated with topical nerve growth factor demonstrated an earlier and faster recovery of corneal sensitivity after LASIK [94]. Finally, in cases where additional factors are contributing to the dryness, such as meibomian gland dysfunction or anterior blepharitis, treatment should include lid hygiene with scrubs and hot compresses and topical azithromycin or oral doxycycline should be considered as well [62].

## 10. Conclusion

Dry eye continues to be the most frequent complication of LASIK, occurring in nearly all patients and fortunately resolving in the vast majority within the first postoperative year. From our experience, preexisting dry eye is the most significant risk factor for severe and prolong postoperative dry eye; therefore, careful preoperative evaluation for signs and symptoms of dry eye is crucial for identifying patients who are prone to develop severe dry eye postsurgery. A thorough preoperative expectation management is to be done, and patients should be advised that they have a significantly increased risk for developing long standing and even chronic post-LASIK dry eye, which could potentially influence the refractive outcome. Other factors that could influence dryness post-LASIK are controversial: for example, it is possible that patients requiring high refractive corrections are at an increased risk for developing chronic dry eye and regression. Enhancement surgery may exacerbate chronic post-LASIK dry eye and therefore should be considered with caution in patients with chronic dry eyes. The ocular surface and tear film must be well managed before the surgery and for several months afterwards. This should be done with stepwise management first with preservative-free artificial tears and, if needed, with the use of topical cyclosporine A, topical autologous serum tears, and punctal occlusion. Treatment is important not only to alleviate ocular discomfort and dryness but also to improve vision quality and refractive results.

## Figures and Tables

**Table 1:** Preoperative dry eye evaluation.

OSDI questionnaire
Slit lamp examination: eyelid disease, tear volume, tears debris, conjunctiva and corneal injection, and fluorescein staining
TBUT
OPI
Schirmer testing

OSDI: ocular surface disease index; TBUT: tear break up time; OPI: ocular protection index.
